# From individual coping strategies to illness codification: the reflection of gender in social science research on Multiple Chemical Sensitivities (MCS)

**DOI:** 10.1186/s12939-014-0078-2

**Published:** 2014-09-10

**Authors:** Geneviève Nadeau, Katherine Lippel

**Affiliations:** School of Political Studies, University of Ottawa, 120 University, Room 7005, Ottawa, Ontario K1N 6N5 Canada; Canada Research Chair on Occupational Health and Safety Law, Faculty of Law, University of Ottawa, 603 King Edward Street, Ottawa, Ontario K1N 6N5 Canada

**Keywords:** Gender-based analysis, Environmentally-induced disabilities, Environmental health, Multiple chemical sensitivies (MCS), Social sciences, Research methodology

## Abstract

**Introduction:**

Emerging fields such as environmental health have been challenged, in recent years, to answer the growing methodological calls for a finer integration of sex and gender in health-related research and policy-making.

**Methods:**

Through a descriptive examination of 25 peer-reviewed social science papers published between 1996 and 2011, we explore, by examining methodological designs and theoretical standpoints, how the social sciences have integrated gender sensitivity in empirical work on Multiple Chemical Sensitivities (MCS). MCS is a “diagnosis” associated with sensitivities to chronic and low-dose chemical exposures, which remains contested in both the medical and institutional arenas, and is reported to disproportionately affect women.

**Results:**

We highlighted important differences between papers that did integrate a gender lens and those that did not. These included characteristics of the authorship, purposes, theoretical frameworks and methodological designs of the studies. Reviewed papers that integrated gender tended to focus on the gender roles and identity of women suffering from MCS, emphasizing personal strategies of adaptation. More generally, terminological confusions in the use of sex and gender language and concepts, such as a conflation of women and gender, were observed. Although some men were included in most of the study samples reviewed, specific data relating to men was undereported in results and only one paper discussed issues specifically experienced by men suffering from MCS. Papers that overlooked gender dimensions generally addressed more systemic social issues such as the dynamics of expertise and the medical codification of MCS, from more consistently outlined theoretical frameworks. Results highlight the place for a critical, systematic and reflexive problematization of gender and for the development of methodological and theoretical tools on how to integrate gender in research designs when looking at both micro and macro social dimensions of environmental health conditions.

**Conclusions:**

This paper contributes to a discussion on the methodological and policy implications of taking sex and gender into account appropriately in order to contribute to better equity in health, especially where the critical social contexts of definition and medico-legal recognition play a major role such as in the case of MCS.

## Introduction

A significant body of research has highlighted the nature and effects of sex-discriminatory policies on men’s and women’s health. Such work underlines the importance of questioning similarities and differences among and between men and women in order to better understand and improve their differentiated health outcomes [1-3]. This sensitivity has led to the implementation of major policy initiatives to encourage or mandate policy-makers to use available data and analysis that connect sex and gender with health issues [4]. The World Health Organization’s *Gender Strategy* [5] and *GBA+*, the Canadian federal policy on gender-based analysis in policy-making across public administration [6], are two key examples of institutionalized frameworks in that regard. The development of this type of policy lens has been criticized for its “simplistic differentiation between categories of men and women” [4], p.11 and the lack of consideration of gender and more complex interactions with factors such as age, socio-economic status, sexuality and ethnicity. In 2000, the General Accounting Office of the United States stressed the need for original data integrating sex and gender in order to produce more timely and relevant policy recommendations [4].

Yet, as Schofield [7], p. 203 underlines, “how policymakers understand gender and health depends very much on how it has been examined, analyzed, and discussed by researchers”. Therefore, the integration of sex and gender in the formulation of research questions and the design of studies plays a key role in the determination as to whether a problem is deemed worthy of policy attention and in the type of response generated.

Major theoretical contributions in the science-policy conversation have come from disciplines in the social sciences. Key examples are Oakley’s sociological differentiation of sex and gender [8], Eichler’s sociological critique of the androcentrism of research theories and methods deemed transferable to women [9] and Butler’s critical philosophical theorization of gender performativity [10]. However, while extensive knowledge about the social, economic and individual determinants of men’s and women’s health has progressively developed, the methodological and theoretical choices made by social researchers looking at health conditions associated with physical environments remain unclear.

In order to explore this question, we reviewed the social science literature on Multiple Chemical Sensitivities (MCS), a contested and disabling diagnosis said to be related to multiple, chronic, low-dose everyday exposures to a cocktail of chemical substances in domestic and workplace environments. Women are reported to be disproportionately affected by this condition. As underlined by Arbuckle [11], both biological and contextual factors explaining sex and gender-specific responses to *acute* chemical exposures have been highlighted in the scientific literature. Biological factors include sex differences in absorption, transport, metabolism, storage and excretion of chemicals following a given exposure, while contextual dimensions include the gendered nature of specific occupational tasks and of protective behaviors and equipment [11]. However, sex and gender-specific responses to chronic chemical exposures are still to be fully understood, which explains the choice of MCS as a case study.

In this article we examine published empirical and theoretical work on MCS, focusing on the characteristics of published papers and the types of study designs associated with different types of gendered perspectives, including those that are blind to gender issues. We conclude with a discussion on potential avenues for integrating gender in research focused on equity in health and, more broadly, in health-related work in the social sciences.

### MCS as a contested diagnosis

An estimated 2 to 6 percent of the population living in industrialized countries has been medically diagnosed, in recent decades, with a toxicant-induced loss of tolerance to very low levels of exposure to chemicals in our day-to-day environments [12]. This sensitization often occurs through a two-stage process, after an initial acute chemical exposure (e.g. pesticides, organic solvents, paints, etc.) often attributed to the workplace [13]. As the sensitivity unfolds, lower and lower levels of “normal” exposures to products and substances (e.g. fragrances, products derived from petrochemicals, pesticides, car exhaust, smoke, etc.) trigger a broad range of symptoms (rhinitis, nausea, modification of heart rhythm, dizziness, etc.) that manifest in multiple organ systems (nervous, respiratory, digestive, etc.) and that vary greatly from one person or exposure to another. This loss of tolerance may degenerate into a chronic condition, which is more or less disabling and life disrupting depending on the person’s “biochemical individuality” [14], on access to proper treatment and on chemical-avoidance and coping strategies. It also often overlaps with other conditions such as fibromyalgia, chronic fatigue syndrome, electromagnetic sensitivity and sick building syndrome [15].

Since the 80’s, MCS has been discussed in numerous workshops and conferences, and recognized both as a disease (Germany, Austria) [16] and as a source of disability (Canadian Human Rights Commission) [17]. Yet, a long-standing controversy remains on the etiology (physiological versus psychological) of the illness, and the World Health Organization [18] has not attributed a specific illness code to MCS in its *International Classification of Diseases* (ICD-10), classifying hypersensitivity under the category “Unspecified effects of external causes/Allergy, unspecified” (T.78.4).

This controversy over the etiology of MCS is still reflected in the diversity of labels used to name the illness. These labels are not merely names: Most of them are underpinned by the exercise of power over the framing of the nature, causes or mechanisms of the condition. They thus are “highly contingent” [14] discursive tools mobilized by specific actors in the debate surrounding MCS, that percolate through social institutions and notably affect processes and outcomes of workers’ compensation issues and health insurance coverage [14]. The chemical industry, for example, has been a strong proponent of the use of the expression “*Idiopathic Environmental Intolerance*”, which evacuates the “chemical” reference to impute the cause to the lack of tolerance of the individual, while some clinical ecologists and environmentally ill persons reject the word “sensitivity” and use the alternative framing of “*chemical injury*”, thereby positioning themselves as victims of the industrial overload from a chemical dominant culture [19]. For the purpose of this paper, we chose to use the term “multiple chemical sensitivities” (MCS) as it is the most widely used in the different types of literature, including in governmental policies, and it is the label used in the 1999 Consensus on MCS [20].

In a “no code, no care” political, administrative and legal context where provision of medical care, social support as well as access to disability insurance and workers’ compensation are strongly dependent on its recognition by national health care systems, in turn influenced by WHO’s ICD [21], the enduring social and biomedical controversy on MCS has so far limited the diagnosis, treatment, and support of environmentally sensitive individuals [22]. Moreover, normative and jurisdictional entities such as courts and physicians’ offices have been sites for dispute on MCS etiology and effects. Numerous authors underline the hostility of those locations that have been known to be sources of stigma, marginalization and social pressure directed towards those who are ill as well as towards empathetic health professionals [23-25]. Dumit [21], p. 578 labeled MCS as one of the “illnesses you have to fight to get”.

The question of gender is at the heart of the issue of equity in health for MCS sufferers, acting as a determinant of access to proper diagnosis, care and social support. Literature suggests that women constitute 60 to 80 percent of persons suffering from MCS [15]. Nicolakakis et al. (on file with authors) showed that work on MCS from the biomedical sciences mainly focused on settling the enduring controversy over its etiology, and only weakly integrated sex/gender. Some broad sex-based hypotheses have however been put forward to explain the disproportionate number of females suffering from MCS, such as interaction of chemicals with estrogens, sex-specific detoxification pathways and proportion of body fat [23]. Gender-based hypotheses are related to the social division of domestic and non-domestic work (e.g. “pink collar jobs”, greater use of cleaning products, etc.) [19,23] and gendered exposures to fragrances and chemicals [11,19,23,26]. Willingness to report symptoms may also be influenced by gender [27]. In this context, what methodological and theoretical pathways do social science researchers privilege when looking at MCS?

## Methods

This study was based on a critical descriptive examination of the social sciences literature, inspired by previous work on scoping reviews [2,28]. An extensive search was made of the social science literature through *Scopus* and *Science Direct* electronic databases. We also hand-searched key peer-reviewed journals related to health and policy to identify potential missing articles. Hand-searched journals included *Medical Anthropology Quaterly*; *Law & Policy*; *Qualitative Health Research*; *Social Science & Medicine*; *Social Theory & Health*; *Sociology of Health & Illness*; *Sociological Inquiry*; *Environmental Health Perpectives*; *Policy Analysis*; *Policy and Politics*; *Policy and Practice in Health and Safety*; *Policy Perspectives*; *Policy Review*; *Policy Studies*; *Political Studies*; *The Political Quaterly*; *Politics & Policy*; and *Politics & Society*.

The initial searches were performed in February and March 2011. In July 2012, we ran new searches in *Web of Science* (including *Social Sciences Citation Index*) with the same keywords in order to validate and update the initial searches. Four papers were added. For each of these databases and journals, the following keywords were used: environmental illness*, environmental sensitivit*, hypersensitivit*, hypersensibilité*, Multiple Chemical sensitivit*, *MCS*. As suggested by Arksey and O’Malley [28], cross-referencing and citation searches were also done by systematically checking the bibliographies and citation data of relevant papers. A saturation point was reached where no relevant new references were identified. No ethical approval was required for this study.

### Criteria of inclusion and exclusion

In order to be included, empirical and theoretical reports had to have been published in English or French in peer-reviewed journals before 2012, and address primarily MCS-related issues through research questions and/or methods belonging to the realm of the social sciences. We chose to include academic theses, as they are also the product of a peer-review process.

In order to avoid overlap with our ongoing parallel review of the research produced in the health sciences, we excluded work specific to psychology and psychiatry from the purview of this study, although these fields generated abundant work on the potential etiology of MCS. For the same reason, we also excluded legal case studies and analysis e.g. [29,30]. Clinical research on issues such as real or perceived efficacy of treatments [31,32] and assessments of occupational performance of individuals with MCS e.g. [33] were also rejected. Finally, we excluded papers addressing MCS regulatory questions and social policy published in regulatory toxicology journals but that did not contain a social science research design or undertaking e.g. [27,29,34].

Similarly, we excluded papers referring to issues related but only peripheral to MCS, such as overlapping illnesses (e.g. electrosensitivity, fibromyalgia, chronic fatigue syndrome, Gulf War Syndrome, etc.). We do however acknowledge the significance of these related conditions and hope to include them in our further examination of research and policy responses.

### Analytic strategy

Full papers corresponding to inclusion and exclusion criteria were retrieved and reviewed. An *Endnote* library was constituted as a data management tool. For every paper, we systematically searched for the following keywords: *sex*, *gender*, *women*, *woman*, *men*, *man*, *girl*, *boy*. Their French equivalent was also searched. In addition, since substantive references to sex and gender might be attributed to other words (e.g. pregnancy, masculinity, etc.), we reviewed every paper in its entirety, looking for implicit content.

Following Greyson et al. [2], p. 2, we developed a “standardized extraction and classification template” for the coding of the included papers of our sample. This consistent categorial template allowed us to identify each article’s research design, keywords, contextual and substantive mention of sex and/or gender, as well as information on the author.

We then further examined each paper to determine the way sex and gender issues were introduced, defined, and treated and how those issues were integrated in the research design (e.g. mention of the composition of the study sample), in the analysis and in knowledge transfer (e.g. data presented by sex). The purpose of the study was not to synthesize findings from the literature, and thus no attempt has been made to highlight the weight of evidence or to assess the quality of the data and analysis presented [28].

We did not find relevant guidance in the literature as to what to consider as an effective *integration* of sex or gender dimensions, even less so in relation to issues of equity in health. This kind of appraisal therefore was a challenge in itself [1]. A common theme to broader ongoing conversations on sex- and gender-based analysis is the location of main concepts along a complex biological and social continuum. Such concepts tend to overlap, rather than being simple binary terms [35,36]. These considerations were however absent from reviewed papers. To develop a finer understanding of the way aspects of *gender* were more or less explicitly integrated in relation to MCS, we used four analytical, interacting categories outlined by leading researchers in sex- and gender-based health research [35,36]. First, *gender identity* pertains to the dynamic and contextually defined ways individuals perceive themselves along a socially prescribed continuum between male–female poles [35]. Associated with it are crucial aspects of the individual’s self, such as aspirations, body image and social roles [35]. Second, *gender roles* refer to more or less sharply defined and differentiated societal norms of behaviour that are enacted at different levels in the day-to-day organization and life experience of both males and females [35]. Third, Johnson et al. [35], p. 4 define *gender relations* as referring to “how individuals interact with and are treated by others, based on their ascribed gender” and to other interacting characteristics such as ethnicity and socio-economic status. Bottorff et al. [37] identify sub-categories to gender relations such as gender interactions, partner communication, femininities and masculinities. Finally, *institutionalized gender* encompasses the complex differentiated distribution of power between genders within societal institutions such as the media, regulatory bodies, the educational system and the medical care network [35]. By producing social norms, expectations and opportunities and by exercising social control, both at micro and macro levels, such institutions are major potential vehicules of gendered health disparities [35].

In light of these considerations and the suggestions of Johnson et al. [35], we did not consider that papers that only *mentioned* sex or gender when describing the composition of the research sample had integrated sex and/or gender dimensions.

## Results

A total of 25 papers published between 1996 and 2011, including two academic theses [23,38], were retrieved and analyzed. In light of criteria previously described, we considered that 13 of them integrated sex and/or gender to some extent.

The following presentation of results is twofold. We first outline the respective methodological and theoretical profiles of the papers that did not and that did integrate sex/gender dimensions related to MCS. Second, we outline the types of integration of gender in the latter papers.

### Methodological and theoretical profiles

#### Journal and authorship profiles

A broad trend emerged from this overview, as papers integrating sex or gender dimensions were dominantly published in gender- or women-oriented journals, while papers where sex and gender dimensions were completely absent were mostly published in journals with a dominant health care orientation, with some exceptions (e.g. *The Canadian Geographer*; *Disability & Society*). The two doctoral theses, presented in social work and geography programs at the University of Toronto, also integrated some aspects of sex and gender. Some journals published both types of papers (*Disability & Society*; *Journal of Clinical Nursing; Medical Anthropology Quarterly; Qualitative Health Research*).

Female authorship largely dominated reviewed literature related to MCS in the social sciences. Ten women and two men were principal authors of reviewed papers, with one scholar (P.R. Gibson) being the main author of a third of them (N = 8). Co-authors were also principally women (N = 13), with only four men being co-authors. In total, 23 authors out of 29 (79%) were women. The latter authored all papers integrating gender dimensions to some extent. Two of them presented themselves as environmentally sensitive [39,40]. In some cases (e.g. P.R. Gibson), authors could be found both in articles that integrated sex/gender dimensions and in articles that did not.

#### Research designs

Studies were primarily conducted in the United States, and some took place, at least partially, in Canada [23,38-45] and in Australia [22,25,45]. Most reviewed papers relied mainly on qualitative research methods, and size of sample varied greatly (N = 1-445).

Studies integrating sex/gender dimensions were articulated around structured, semi-structured or open interviews [13,23,38], illness narratives [42], unstructured life histories [40], photo-elicitation [23], qualitative questionnaires [42,46,47], quantitative measurement tools [48,49] as well as MCS listserves and chatrooms [14,44].

Papers where there was not a sex/gender dimension were not methodologically dissimilar, and relied mainly on case studies [25], participant observation [25,39,43], interviews [21,25,39,43,50], focus groups [51], quantitative measurement tools [51,52], weblist or newsgroup analysis [21] and textual analysis [25,43]. Three papers were not explicitely based on empirical data [14,19,53].

Participants were generally persons with MCS, although some authors also interviewed or surveyed MCS patients’ family members [43,45], health and social service professionals [22,23,25,38,41,42,45,52], legal practioners [22,45], political representatives [22,45] and MCS advocates [22,45]. Women constituted between 0 and 100 p.cent of the research sample, but represented more generally 80 to 90 p.cent of it. One paper [53] was structured around a case-study of an injured (male) worker, although this choice was not further contextualized. Some papers justified the greater inclusion of women in study samples by the higher reported prevalence of MCS among women [23,40,47]. Women’s roles of care giving, homemaking and childbearing as well as greater use of domestic and care chemical products were also put forward to introduce a focus on women [40]. One author [41] highlighted that women were “highly represented” in her sample (90 p.cent), although the study was not focused on women, hereby reproducing a “typical gender split” in environmental illness prevalence.

Although the majority of papers included men in their sample (when the sex of sample composition was mentioned), issues specifically experienced by men suffering from MCS were discussed in only one paper [19]. Interestingly, that paper flows from an explicitly feminist standpoint. In contrast, in another article, although Gibson et al. [13] included 25 men in their study of 203 participants on identity changes associated with MCS, none of the reported quotations in the paper were attributed to males, and substantial parts of the paper were women-focused, without explanation.

#### Purposes of the studies

Papers integrating gender insights to some extent appeared more focused on the personal capacity and propensity of the women suffering from MCS to engage with the social context (agency), and generally emphasized how these women develop an ability to manage their condition. This was achieved by documenting the life experience of environmentally ill persons with regard to experiences of stigma associated with the contested nature of the diagnosis [19,24,40,44], isolation [40] identity issues and life disruption [13,46], including their adaptation to this “corporeal (embodied) chaos” [23], p. 64 via the creation of “safe spaces” [23]. Papers focused on sources of social support and hope [48] as well as on issues of work accommodation and compensation [24,49], MCS activism [14,19,23] and community access [47] also generally integrated gender. In three papers, a gendered perspective of the lived experience of *women* with environmental sensitivities was at the core of the analysis [23,24,40].

Papers that did not integrate a gender perspective also discussed issues such as coping strategies [39], impacts of MCS on isolation [50] and life trajectory [51,53]. These papers however further addressed broader issues surrounding MCS, such as illness legitimation and diagnostic decision-making strategies [52], legal recognition and medical codification of MCS [22,25,45], dynamics of expertise and decision-making in workers’ compensation claims [22,25,45], legitimacy [22,25] and professional stigmatization of empathetic MCS experts [45]. Also discussed were the sense of place and illness experience associated with MCS local “outbreaks” [43], p. 87 and technology-related disability barriers [54].

#### Theoretical standpoints

Papers in our sample did not link, in general, empirical data to a strong theoretical framework. However, some articles integrating sex and/or gender dimensions located themselves more or less explicitely within feminism [19,38,46], ecofeminism [19,38,40], social constructivism and phenomenology [14], feminist disability theory [38], risk society theory [41] and geography of health’s theory of corporeal chaos [23,41].

Some interesting concepts, having repercussions for equity issues, were brought jointly to a discussion integrating gender, such as the historical psychologization and delegitimization of women’s health issues [14,19,24,38,41,44,47], the misunderstanding and vulnerabilities associated with hidden disabilities [45] and the sex bias in environmental and occupational health research [19,23,40,41]. Murphy [14], in her work on biochemical individuality with a gendered relationship to risk culture, was amongst those who more finely articulated sex/gender theorization with the actual issue of MCS.

Papers where sex and/or gender dimensions were absent generally presented a more specifically developed theoretical component, by proposing, for example, an epistemological discussion of power and biomedical uncertainty [45] or by addressing the strategies of diagnosis, codification and expertise of contested illnesses [21,45,52]. Critical impairment and disability studies were also mobilized [54]. Fletcher [43] developed a phenomenological analysis of the sense of place for persons with MCS as an equivocal illness. Coyle [41] applied the complexity theory to health and health care issues to develop, in the absence of a biomedical or social consensus on the etiology of MCS, an alternative epistemological conception of symptoms and lived experiences pertaining to environmental sensitivities as a complex and emergent illness. Other papers presented an essentially descriptive account of empirical data, without further theoretical framework [39,50,53].

### Use and integration of sex and gender language and concepts

Reviewed papers integrating textual references to sex and/or gender did not make distinctions between these two concepts, with the exception of Coyle [23,41]. References to sex were made relatively to the sex-segregated statistics on prevalence of MCS [19,23,24,41,44,46]. Other studies [48,49,51] presented quantitative results by “gender”, in some cases underlining the absence of significance in differences between genders in scores [48,49]. No finer gender analysis was however drawn from such segmentations.

As for the implicit or explicit integration of *gender* dimensions in social science papers on MCS, we can divide them according to the four categories identified by Johnson et al. [35,36] and outlined above.

1. *Gender roles* of women were at the core of gendered perspectives in the reviewed literature. As mentioned above, gendered roles associated with specific chemical exposures and continued use of domestic cleaning products were brought forward by some authors [19,23,24] to contextualize the higher prevalence of MCS among women. These exposures take place in a gendered nexus between bodies and built environments such as kitchens and offices [14].
  Furthermore, women with MCS may feel unable to perform gender-normalized roles such as that of a devoted spouse and/or mother and sexually attractive and active being [13,41]. Female participants’ feelings associated with the failure to meet their own expectations in the performance of such roles, such as guilt, shame and anger, were explored by some authors [40]. Changes in the values of women with MCS regarding these gendered roles, such as expectations regarding the use of cosmetics and personal care products for the sake of physical appearance, were also reported [40]. Gibson [19] underlines that deviance by women from these prescribed roles is socially labeled as maladaptation. Self-care in the context of MCS can be reached through either rejecting or reconfiguring culturally gendered roles related to house-keeping to secure an exposure-free environment [14]. It can also require redefinition of health care. Gendered performance of self-monitoring and micromanagement of the body (diets, detoxification, vitamins, etc.) are good examples [14].
  In the only mention of issues specifically experienced by men with MCS, Gibson [19] reports that environmentally sensitive men somehow break a gender bargain by acknowledging their vulnerability to external factors, for example by wearing a respiratory mask, and by assuming their special needs to stay in clean workplaces or public environments. Both behaviours are perceived as unmasculine.
2. These gendered roles are intrinsically linked with *gender identity*. Chemical avoidance implies the replacement of fashionable clothes, aesthetic household items and common personal care products and habits associated with the social construction of femininity or masculinity by often less attractive substitutes. Gibson et al. [13] reported that women with MCS might briefly adopt risky behaviours such as wearing conventional make-up in order to preserve their sense of femininity by conforming to cultural standards of physical appearance. This link between femininity and potential health outcomes has been developped already in the gender and health literature [37]. Also, in a different approach to gender identity and MCS, Murphy [14] highlights that white, middle-class women with MCS have been prompt to develop gendered “victim identities”, a label supported by white feminist scholarship. For Murphy [14], p. 99, such identity appears “not equally available, nor as viable” for racialized men and women who clean commercial workspaces and upper-class houses, for example.
3. Different axes of analysis were also located in the realm of *institutionalized gender*. Some authors underlined the traditional diagnosis substitution and trivialization of women’s health matters by medical institutions (manuals, procedures, etc.), notably through psychological and psychiatric labeling of typically female illnesses [14,19,23,38,41,47] and differential treatment of men and women [19]. Murphy [14], p. 88 echoes this legitimacy issue by asserting “MCS is removed from the realm of legitimate corporal illnesses and becomes instead a gendered expression of psychological distress”. Coyle [41] and Gibson [19,47] remind us that the 19th century’s diagnosis of hysteria was attributed primarily to upper-class women, who incidently also constitute the dominant profile of chemically sensitive persons, accused of seeking attention as well as work avoidance and compensation. Gibson [19], p. 485 thus reports that women are “held hostage by professionals who seek to quell, study, and label them as their bodies speak in a language not decipherable within an old paradigm”. An environmentally sensitive woman in one of the studies was thus told by her physician to get wine and chocolates, as well as a walk in a park in order to relieve symptoms [19]. These misconstructions, concurring with what Murphy [14], p. 89 terms a social “abjection” of MCS, raise health equity issues as they have potential effects on the improvement of the health and well-being of the person, by delaying access to proper diagnosis and multiplying harmful treatments in the interim [14].
  Other institutional aspects were also identified. Coyle [41] put forward the toxicological and health research bias where the male body is empirically taken as the standardized norm, while assuming that conclusions are sexless and genderless. Gibson [19] underlined the gendered division of labour as it is socially institutionalized, suggesting that women are more often confined to low-wage clerical jobs characterized notably by a lack of control on poor air quality and continuous exposures such as photocopy fumes and fragrances.
4. Dimensions of gender roles, gender identities and institutionalized gender all relate to *gender relations* at both micro and macro levels. More specifically, power dynamics at play between typically male physicians with diagnosis authority and female MCS patients in a position of vulnerability were underlined in reviewed papers. Such dynamics took the shape of trivialization of their health problems [46,47], and female participants in one reviewed study reported the imperatives of emotional restraint, objectivity and conformist appearance in order to be legitimized by health professionals [19].

## Discussion

### Making the choice of a gender lens: a matter of policy strategy?

Results showed that work on MCS in the social sciences that integrated a gender lens tended to focus on gender roles and identity. Complex and often costly coping strategies to diminish disabling exposures and recover health appear to be the only possible avenues for the environmentally ill (e.g. accommodation with relatives and neighbors, withdrawal from the workplace, creation of a safer housing environment, alternative treatments, etc.) [31,32,39]. These new social practices are mainly located outside of public spaces, and thus remain largely invisible and lead to a certain civic disconnection of those affected, sometimes identified as “bubble people” [44], p. 203. In that regard, the social sciences seem to have a role to play to develop a better understanding of how their condition is experienced by those suffering from MCS.

However, as underlined partially by Gibson [47], such a narrative perspective runs the risk of embedding MCS in a paradigm of individualization of risk characterized by an emphasis on personal strategies of adaptation and “precautionary consumption” [55], moving further away from more upstream, contextualized considerations on the chemical contamination of the domestic and work environments. The equity concerns raised by such a risk could drive a more reflexive operationalization of sex- and gender-based analysis [1]. In the case of social science research on a contested condition such as MCS, this shift could translate into a greater stream of studies moving from a dominant focus on women’s agency to a gender-sensitive perspective addressing, in theoretically and methodologically compelling ways, more macro and systemic problems such as diagnosis dynamics and illness codification in formal policies. It could also lead to a more sensitive analysis of work environment issues, an analysis that could reveal differential exposures of men and women to workplace contaminants specific to their respective work environments [56].

Our results also raise questions about the choice of *not* explicitly integrating gender in the study of socially and medically contested health issues such as MCS. On one hand, it is at least legitimate to discuss whether a strong gendered focus on women’s individual coping experiences might, in the context of a contested environmental illness, open the door to traditional delegitimization bias related to women’s health, and therefore impede the goal of advancing MCS as a public health matter relevant to the policy agenda. In that regard, it could well be appropriate that key papers such as Phillips’ [22] or Swoboda’s [52] did not focus on gender in order to highlight more systemic factors. Perhaps an article designed to inform policy-makers may legitimately, for strategic reasons, choose to obfuscate gender dimensions if the targeted readership is itself averse to gender sensitivity. On the other hand, and as outlined previously, recent developments, perhaps still marginal but yet invigorating, promote new gold standards of integration of sex and gender in health-based research, and it is the responsibility of social science researchers to embody these best practices in order to produce the best scientific knowledge possible.

Such questions bring to the forefront the importance of handling approaches accounting for sex and gender with a sensitivity to “unanticipated consequences” [57], p. 57 potentially unfolding from such a focus [57], as well as the relevance of building on innovative gender-based theoretical and methodological frameworks. Among them, sex and gender-based analysis (SGBA) is “an approach to research and evaluation which systematically inquires about biological (sex-based) and sociocultural (gender-based) differences between women and men, boys and girls, without presuming that any differences exist” [58]. If such an endeavor could indeed contribute to elicit finer gender dimensions related to MCS along a complex conceptual continuum, it could also help us to highlight “how biological processes defined by sex combine with gender to influence systemic outcomes that, in turn, reinforce subsequent downstream physiological events” [57], p. 51.

### Framing gender with sensitivity: the challenge of methodological coherence

The conflation, in some of the reviewed papers, of the term “gender” with the sole reference to women raises analytical issues of concern. Some articles reported only women’s quotations and challenges while claiming to report on the experience of “persons with MCS”. Greaves [4], p. 7 hypothesizes that such a tendency might be explained by a will “to mask a focus on women when that proved unpopular politically”. However, this conflation of women and gender both clouds women-specific issues related to environmental sensitivities, by not naming them explicitly, and fails to provide a comprehensive perspective on the specific realities of men with MCS, who constitute a non-negligible 20 to 40 p. cent of persons with this condition [15]. In our review, when men were included in the research sample of a study, quotations from them or references to their specific realities were rarely reported. This is all the more surprising since the only work that specifically reported on hypersensitive men’s perspectives showed that they also experience stigma and trivialization, although on a different basis related, among other things, to masculinity [19]. Moreover, some papers were strictly focused on women with MCS, but did not engage with a gender-sensitive analysis, even though the proposed analysis of body- and environment-centred techniques of creation of a “safe space” might have been extended to men with MCS as well. Were such a methodological choice to have been made, it could have led to a better differentiated understanding of commonalities and differences in the sexed and gendered issues dealt with by men and women living with MCS.

Thus, our results echo the conclusion of Messing et al. [56] on the importance of not assuming *a priori* that certain questions apply to only one gender. Actual conversations about the integration of sex and gender in health research support the importance of systematically questioning whether it is justified or not to focus on only one or on both sexes [56], or on how sexes and genders connect or interact in the production of specific health outcomes [57]. Such a stance however does not exclude the relevance of focusing a study or a paper on women, as can be noted from the significant contributions of reviewed papers.

### Problematizing differences: an actual challenge for the social sciences

Our results highlight the lack of a fine problematization of differences 1) *between* men and women, as well as 2) *within* each gender group.

1) The discourse on the prevalence of MCS is a good example of the first case. Some papers [14,19,32,40,47] mentioned that more women than men are environmentally sensitive. Others [23,24,38,41,44,46] also relayed statistics according to which women purportedly represent 60 to 80 p.cent of the persons suffering from this condition. However, this differentiation was generally presented as one fact among others, and was poorly contextualized.

This silence is surprising since the major role of the social sciences is to underline the importance of questioning what might otherwise appear as a given. Feminist literature, for example, contributed to a major extent to question and deconstruct the naturalization of differences between men and women [59]. Thus, it appears important to raise questions related, for example, to gendered dynamics of diagnosis and interactions with health systems, from the perspective of both men’s and women’s health: Would men be more likely to automatically receive a diagnosis of acute occupational intoxication for similar symptoms labeled as MCS in women? Are women asked at all by their physician if their professional activities expose them to chemicals? Other factors, such as differences in risk perception, socialization and propensity to seek treatment [56] can also have a significant impact and are worthy of examination. Classic studies from the health literature, such as work on the overmedicalization or dismissal of women’s health issues, might also contribute to strengthen theoretical analysis in this regard. So could studies on masculinities and the ways gendered expectations may lead to acceptance by men of physical job exposures and demands that must be endured regardless of their impacts on health.

2) As for the lack of problematization of differences *among* men and *among* women in the reviewed literature, we were surprised to see how women were referred to as a homogeneous group, and how other interacting social and personal characteristics were rarely taken into account. An exception in reviewed work is Murphy’s analysis [14], p. 108, which suggests that the focus on “safe place” rather appears as part of a “gendered, raced, and classed relationship to late-capitalist ‘risk’ culture” and to built environments.

Women do not constitute a homogeneous group; nor do they all have the same domestic and occupational chemical exposures [11]. The same is true of men, whose occupational exposures vary greatly depending on factors related to socio-economic status, racialization and ethnicity. There are problems associated with an unreflexive implementation of gender-focused frameworks that homogenize men’s realities and women’s realities, and thus neglect other key factors impacting health [57,60].

Therefore, there is still opportunity to more appropriately identify *which* men and women tend to face which issues related to MCS or to receive an MCS diagnosis, and why. This appears important in order to make sure that a focus on gender does not obfuscate characteristics such as ability, ethnicity, “race”, age, sexuality, and socio-economic status, underpinnings of other dimensions of inequality. Does better access to medical and environmental health information and to financial resources explain why more Caucasian clerks than immigrant cleaning ladies, possibly more exposed to a cocktail of solvents and chemical products, get both a voice and a diagnosis related to MCS? Is MCS less likely to be diagnosed or treated when migrant farm workers are exposed to pesticides than when a toxic spill affects unionized blue-collar workers? These questions are part of a finer understanding of the way environmental determinants intersect with other aspects of sex and of the social context of gender to produce specific health outcomes related, in this case, to MCS. In that regard, appropriate research should investigate other biological and social factors, for example those connected to ethnicity or “race”, just as comprehensively while maintaining a sex and gender lens. Such questions are also essential to our understanding of institutionally recognized illnesses and are inseparable from perspectives on recognition, codification, legitimacy and expertise, notably from a workers’ compensation policy perspective, since initial triggering exposures of MCS reportedly often occur in workplace settings [12]. Finally they are also intrinsically related to the understanding of gendered entries into the “sick role” [21,61], notably in relation to perceived expected performances of masculinity in the role of a productive worker.

Of interest in this regard are Krieger’s [62] concept of embodiment as well as the intersectionality framework [63], which emphasize the interrelation, in the production of health and determinants of health, between sex, gender and other micro and macro level-factors such as age, income, education, ethnicity, sexual orientation and ability [4,57,64]. This can include a reflective consideration of a researcher’s positionality.

## Conclusion

This paper aimed to explore, through a critical examination of the literature on Multiple Chemical Sensitivities, the methodological and theoretical approaches applied in the social science literature to a contested environmental issue with a strong gender component.

Results highlighted that papers integrating sex or gender dimensions were dominantly authored by women, comprised a large majority of women in the research samples and were published in gender- or women- oriented journals. These papers mainly focused on the personal capacity and propensity of the women suffering from MCS to develop an ability to manage their condition and engage with the social context. Papers that overlooked sex or gender dimensions generally addressed broader social issues such as the dynamics of expertise and the medical codification of MCS, and they did so from more consistently outlined theoretical frameworks. Papers integrating gender mainly did so by discussing *gender-normalized roles* of women associated with specific chemical exposures as well as the social construction of *gender identity* associated with femininity. Aspects of *institutionalized gender* and *gender relations* were more marginally discussed. Importantly, although men constitute 20 to 40 percent of persons suffering from MCS [15], only one paper mentioned issues experienced by men suffering from MCS [18], and none explored the literature on masculinity.

After discussing the choice of mobilizing or not a gender lens, we underlined some methodological inconsistencies ensuing from terminological and thereotical confusions such as the recurring conflation of gender and women. We concluded by pointing to the need for a better analysis of differences both *between* genders as well as *within* each gender group, groups often assumed to be homogeneous.

The lack of clarity in conceptual and methodological research designs regarding sensitivity to sex and gender is not specific to the social sciences [37]. Although some funders such as the *Canadian Institutes of Health Research* and the *U.S. National Institutes of Health* have implemented or are implementing systematic requirements to consider and report the relevance of sex and gender dimensions at the outset of the research design [65,66], the explicit consideration of sex and gender remains marginal in the production and diffusion of most mainstream human health-related research [4]. Examination of the relevance of a sex and gender-based analysis has not yet, for example, been institutionalized as a requirement for mainstream health-related scientific journals [2]. However, much work remains to be done to better understand *how* a gender-based perspective on environmental health research in the social sciences can go beyond the essential yet insufficient examination of differences between males and females in order to account for finer dimensions of sex and gender. We also need to develop a finer understanding of what is lost on the policy level when there is no gender analysis integrated in scientific work, both in the social sciences and the biomedical literature. Yet, major methodological questions remain: What do we consider as “integration” of gender (as a variable; descriptive facts; institutionalized aspects; etc.)? How should we integrate these dimensions in research on more “upstream” aspects of environmental health issues such as policy sciences? Should the answers to these questions vary depending on the phenomenon being studied?

This paper made a case for the unfolding of more systematic, thorough and reflexive efforts to consistently and critically investigate sex and gender dimensions in health-based research, using appropriate and innovative methodological tools and theoretical frameworks. Existing frameworks such as SGBA and intersectionality, discussed above, could well contribute to move social science research beyond current confusions and challenges. These frameworks could better allow researchers to fulfill their essential role in problematizating equity in health. With regard to MCS, the mobilization of innovative frameworks integrating sex, gender and other social determinants of health, including environmental determinants, could contribute to more effective policy conversations on issues such as disability recognition, workers’ compensation and financial support for facilitating chemical avoidance. In the case of MCS, all these questions are essential to the improvement of health outcomes for both men and women.
